# Psychological well-being, mental distress, metabolic syndrome, and associated factors among people living in a refugee camp in Greece: a cross-sectional study

**DOI:** 10.3389/fpubh.2023.1179756

**Published:** 2023-06-16

**Authors:** Florian Knappe, Konstantinia Filippou, Antonis Hatzigeorgiadis, Ioannis D. Morres, Emmanouil Tzormpatzakis, Elsa Havas, Harald Seelig, Flora Colledge, Sebastian Ludyga, Marianne Meier, Dominique de Quervain, Yannis Theodorakis, Roland von Känel, Uwe Pühse, Markus Gerber

**Affiliations:** ^1^Department of Sport, Exercise and Health, University of Basel, Basel, Switzerland; ^2^Department of Physical Education and Sport Sciences, University of Thessaly, Trikala, Greece; ^3^Department of Nutrition and Dietetics, University of Thessaly, Trikala, Greece; ^4^Department of Health Sciences and Medicine, University of Lucerne, Lucerne, Switzerland; ^5^Interdisciplinary Center for Gender Studies, University of Bern, Bern, Switzerland; ^6^Division of Cognitive Neuroscience, University of Basel, Basel, Switzerland; ^7^Department of Consultation-Liaison Psychiatry and Psychosomatic Medicine, University Hospital Zurich, University of Zurich, Zurich, Switzerland

**Keywords:** prevalence, physical health, non-communicable disease, PTSD, stress, migrant, fitness

## Abstract

**Background:**

Forcibly displaced people face various challenges and are therefore at higher risk of being affected by mental and physiological distress. The present study aimed to determine levels of psychological well-being, PTSD symptom severity, metabolic syndrome, and associated factors among forcibly displaced people in Greece in response to WHO’s call for evidence-based public health policies and programs for forcibly displaced people.

**Methods:**

We conducted a cross-sectional study among *n* = 150 (50% women) forcibly displaced people originating from Sub-Sahara Africa and Southwest Asia living in a Greek refugee camp. Self-report questionnaires were used to assess psychological well-being, symptoms of PTSD, depression, generalized anxiety disorder and insomnia, perceived stress, headache, and perceived fitness. Cardiovascular risk markers were assessed to determine metabolic syndrome, and cardiorespiratory fitness was measured with the Åstrand-Rhyming Test of Maximal Oxygen Uptake.

**Results:**

The prevalence of mental distress and physiological disorders was overall elevated. Only 53.0% of participants rated their psychological well-being as high. Altogether, 35.3% scored above the clinical cut-off for PTSD, 33.3% for depression, 27.9% for generalized anxiety disorder, and 33.8% for insomnia. One in four (28.8%) participants met criteria for metabolic syndrome. While the prevalence of moderate or severe insomnia symptoms and metabolic syndrome differed little from the global population, the risk of being affected by mental distress was markedly increased. In multivariable analysis, higher perceived fitness was associated with higher psychological well-being (OR = 1.35, *p* = 0.003) and a decreased likelihood for metabolic syndrome (OR = 0.80, *p* = 0.031). Participants with elevated psychiatric symptoms were less likely to report high psychological well-being (OR = 0.22, *p* = 0.003) and had increased odds for higher PTSD severity (OR = 3.27, *p* = 0.034). Increased stress perception was associated with higher PTSD symptoms (OR = 1.13, *p* = 0.002).

**Conclusion:**

There is an elevated risk for mental distress compared to the global population and an overall high mental and physiological burden among people living in a Greek refugee camp. The findings underpin the call for urgent action. Policies should aim to reduce post-migration stressors and address mental health and non-communicable diseases by various programs. Sport and exercise interventions may be a favorable add-on, given that perceived fitness is associated with both mental and physiological health benefits.

## Introduction

Human-made conflicts and natural disasters have led to a doubling of forced displacement in the past 10 years, reaching an all-time high of 94.7 million affected people in 2021 (1). These figures will likely continue to grow due to armed conflicts, political oppression and environmental changes (2). Even though most people are internally displaced or find refuge in neighboring countries, forced migration to Europe has more than tripled in the past decade (3). Greece has been one of the main entry points for over 1.2 million forcibly displaced people since 2015, as one of the southernmost countries in Europe and due to its close sea border with Asia (1).

Forcibly displaced people are generally challenged with severe mental and physical strains before, during, and after their flight (4). While infectious diseases and injuries are often treated shortly after arrival, new complaints, such as non-communicable diseases, can arise (2). In addition, ongoing post-migration stressors such as uncertainty about migration status, legal barriers, harsh treatment by authorities, socioeconomic hardship, language barriers, discrimination, social exclusion, limited access to health services, and lack of access to healthy food hinder the recovery from pre-migration trauma and increase the risk of being affected by posttraumatic stress disorder (PTSD) (2, 5). While it has been reported that forcibly displaced people show high levels of resilience (6), the likelihood of being affected with mental and non-communicable diseases is markedly increased compared to the host population (7, 8). A recent meta-analysis (9) documented prevalence rates among forcibly displaced people of 31% for PTSD, 25% for depression, and 14% for generalized anxiety disorders. In addition, a series of physical health complaints have been recorded among forcibly displaced people that can be clustered in the metabolic syndrome and are associated with an increased risk for cardiovascular diseases and diabetes (7, 10). Overall, this double burden of mental and physical comorbidities negatively affects the psychological well-being of individuals and families and drastically reduces life expectancy compared to unaffected counterparts (11). Organized sport and exercise activities have shown promising effects in addressing mental and physical complaints (12). At the same time, these complaints are often accompanied by low fitness levels, whereas low fitness levels may contribute to these physical complaints and, at the same time, also be a cause of mental distress and physiological disorders (13).

Prevalence of mental disorders vary widely across studies with forcibly displaced people, ranging from 2 to 88% for PTSD, 5 to 81% for depression, and 1 to 90% for generalized anxiety disorder (14). While heterogeneity in prevalence can be caused to some extent by methodological differences, the discrepancy could also be attributable to sociodemographic and post-migration differences (15). Therefore, it is important to obtain population and context specific data. A recent call has been made by the World Health Organization (8) for more detailed data to accurately monitor and address the health status of forcibly displaced people. This monitoring should also encompass the conjunction of mental and physical determinants (16). Understanding the specific health needs of forcibly displaced people and their circumstances can provide valuable indicators for targeted programs. Timely addressing individual mental and physical challenges could prevent short- and long-term adverse consequences for forcibly displaced people and the host countries. Since the marked increase of forced migration to Europe in 2015, however, only three studies (17–19) have examined the mental health of forcibly resettled adults in Greece. As these studies focused predominantly on one mental condition, a more comprehensive approach that incorporates mental and physical health parameters is needed.

The present study aims to determine levels of mental distress, physiological disorders, and associated factors among people living in a refugee camp in Greece. Specifically, the study explored the prevalence of mental distress and physiological disorders compared to the global population and examined the association of specific socio-demographic characteristics and clinical parameters with psychological well-being, PTSD symptom severity, and metabolic syndrome to identify risk groups and obtain indications for possible interventions.

## Methods

### Design and setting

This analysis is part of a larger randomized controlled trial that examines the effects of a sport and exercise intervention on mental health, cardiovascular risk markers, and physical fitness among people living in a refugee camp in Greece (ISRCTN16291983). The sample and the procedures of the present study were based on the sampling and the procedures described in the registration of the project (20). In this paper, we present cross-sectional data from the baseline data assessment. Ethical approval was obtained by the Research Ethics Committee of the University of Thessaly, ref. approval no. 39 and the ethical review board of Northwest and Central Switzerland, ref. approval no. AO_2020–00036.

The study was implemented in a refugee camp in central Greece. The camp was founded in 2016 and can host around 1700 people. At present, the camp operates under the management of an officer appointed by the Ministry of Migration and Asylum and serves as a temporary accommodation center where people wait for their asylum applications to be processed. People live in the camp in containers, which we will refer to as households in this study. While a family has a container for itself, individuals share the container with up to four people of the same sex and origin. The containers are equipped with a bathroom, cooking facilities and air conditioning. At the time of data collection an adult received 150 Euros per month for expenses such as food, clothing, telephone bills, hygiene items, and public transportation. In case of health complaints, a medical center with two medical doctors, nurses and two psychologists from the Greek national health service provided primary health services. The camp is located in a rural area. The nearest village with a small grocery store is 15 min walking distance. A town with an ATM, post office, clothing store, or the possibility to print documents is 16 kilometers away and can be reached by public transportation. While adults are not allowed to engage in paid work due to legal barriers, school-age children can attend public schools.

Based on the data provided by the site management, 1,376 residents lived in this camp in February 2021. Among them, 920 (67%) residents were aged 16 to 59 years, and 39% were women. The forcibly displaced population is diverse in terms of sociodemographic background. Most residents were from Afghanistan (45%) and Syria (25%), whereas the remaining 30% were from West Asian (11%), Sub-Saharan zone (17%), or other (2%) regions.

### Participants

Eligible to participate in the study were individuals who (a) lived in the selected refugee camp, (b) were between 16 and 59 years old, (c) were able to read in English, Arabic, Farsi, or French, and (d) provided written informed consent. For ethical reasons, a broad age range was defined as an inclusion criterion, in order to enable as many as possible to participate in the sport and exercise activities of the intervention trial. The site management provided a list of camp residents sorted by language. Based on this list, potentially eligible households were screened for sociodemographic background. Recruitment was done by households to avoid exclusion of individuals from the same household. A random sample stratified by sex was finally drawn from all screened and eligible households. Additional households were drawn in case of non-appearance in order for the parent project to have a sufficient number of participants. A minimum sample size of 136 participants was estimated based on a power analysis to detect an intervention effect on PTSD symptoms (20).

### Procedure

The screening, recruitment, and assessment processes were carried out in May 2021. At the beginning of the study, as many households as possible were screened to obtain an overview of the sociodemographic background of the camp population. Residents who were about to participate in the study were asked to provide written informed consent before data collection. Information about the purpose and procedure of the study was provided in writing and verbally. All participants were assured that participation is voluntary and that they could withdraw without any negative consequences, particularly concerning their asylum application. This approach protects participants from potential harm, coercion, and exploitation. However, cultural and language differences may lead to misunderstandings, with false expectations compromising voluntary participation (21). To mitigate such misunderstandings, the study recruited 10 research assistants from the camp residents based on the recommendation of the site management. These research assistants played a critical role in approaching residents, explaining the study, obtaining informed consent, translating, and assisting with data collection.

All measures were taken at the nearby Department of Physical Education and Sport Science of the University of Thessaly due to the availability of necessary facilities and equipment. Participants were informed about their results after the assessment and were referred to a specialist if a health risk was indicated. Participants received further compensation for their participation in the form of a meal and sport equipment. The highlighted measures ensured that the research provided reciprocal benefits for those participating in the study.

### Measures

Trained research staff were responsible for the data collection of the outcomes (psychological well-being, PTSD symptom severity, and metabolic syndrome) and predictor variables (sociodemographic background, symptoms of depression, generalized anxiety disorder and insomnia, perceived stress, headache, anemia, cardiorespiratory fitness, and perceived fitness) by following a standard operating procedure. All questionnaires were provided in English, Arabic, Farsi, and French matching the native language background of most participants. Additionally, translators were present during the data assessment when needed. The measures have been previously used with forcibly displaced people (15, 22–28), have been validated in English, Arabic, Farsi, and French (29–50), and had acceptable or good internal consistency (Cronbach’s alpha >0.7) in our pilot study (51). We used clinically relevant cut-offs to determine prevalence rates of mental distress. A growing body of literature emphasizes that self-report symptom-based measures are likely to inflate the prevalence of mental distress in populations of forcibly displaced people (52–54). Therefore, more conservative cut-off values were chosen (36, 40, 43, 55). As the instruments for perceived stress, headache, and perceived fitness are not used for diagnostic purposes, the classification into high and low profiles was done *via* median split. Information on the sociodemographic background of the participants, including sex, age, origin, educational background, number of relatives in the camp, time fleeing (in months), and time in camp (in months) was collected with a questionnaire.

### Mental health

Psychological well-being was assessed with the five-item World Health Organization Well-Being Index (WHO-5), which is specifically designed to measure mental well-being (29). Each of the 5 items is scored on a Likert scale from 0 (at no time) to 5 (all the time). Items were summed up and then multiplied by 4, resulting in an overall index between 0 and 100. Psychological well-being was finally dichotomized in high (>50) and low (≤50) well-being (56).

PTSD symptoms were assessed with the 22-item Impact of Event Scale-Revised (IES-R) (50). The instrument is internationally accepted and not culturally specific. The IES-R items refer to DSM-5 (57) and ICD-10 (58) criteria of PTSD. Items were answered on a five-point Likert scale from 0 (not at all) to 4 (extremely), resulting in an overall index between 0 and 88 points. The cut-off for a possible PTSD diagnosis is set at ≥46 (55).

Depressive symptoms were assessed with the 9-item Patient Health Questionnaire (PHQ-9) (36). Items of this instrument refer to DSM-5 criteria for major depression. Answers were given on a four-point Likert scale ranging from 0 (not at all) to 3 (nearly every day). The overall index varies between 0 and 27. A score of ≥15 indicates moderately severe or severe depressive symptoms (36).

Anxiety symptoms were assessed with the 7-item Generalized Anxiety Disorder scale (GAD-7) (40). The instrument refers to DSM-5 criteria for generalized anxiety disorder. Participants were asked to rate the frequency of anxiety symptoms on a four-point Likert scale from 0 (not at all) to 3 (nearly every day). The overall index ranges from 0 to 21, with a score of ≥15 being interpreted as more severe anxiety levels (40).

Insomnia symptoms were assessed with the Insomnia Severity Index (ISI) (43), a brief screening measure of insomnia and an outcome measure in treatment research, which takes into consideration the criteria for insomnia of the DSM-5. The instrument contains 7 items, which were answered on a five-point Likert scale from 0 (no problem) to 4 (very severe problem). The overall index is scored between 0 and 28. Values of ≥15 indicate possible moderate insomnia (43).

Perceived stress was assessed with the 10-item Perceived Stress Scale (PSS-10) (46). Participants were asked how often they find their lives overwhelming, uncontrollable, and unpredictable on a five-point Likert Scale from 0 (never) to 4 (very often). The score of the positively stated items (4, 5, 7, and 8) is reversed before summing up all items. The overall index ranges from 0 to 40, with higher scores indicating a higher level of perceived stress.

### Physical health

Cardiovascular risk markers were assessed to determine metabolic syndrome. A flexible tape was used to determine waist circumference. Systolic and diastolic blood pressure was measured after the participant had rested for 5 min while seated. Blood pressure was measured three times within 5 min with an Omron® digital blood pressure monitor. Evidence for the validity of this device has been reported previously (59). The participants’ finger was pricked once for all (capillary) blood analyzes to collect approximately 10 blood drops. One drop was used for the detection of anemia. Thus, hemoglobin (Hb) levels were measured with a HemoCue® Hb 301 system (HemoCue AB; Ängelholm, Sweden). The incidence of anemia was defined as <120 g/L for women and < 130 g/L for men (60). For the assessment of blood lipids (high-density-lipoprotein cholesterol, fasting plasma triglycerides) and average level of blood glucose over the past 3 months (glycosylated HbA1c), blood samples were analyzed with an Afinion 2 analyzer (Abbott, Wädenswil, Switzerland). One drop of blood was taken by the test strip and read by the analyzer. A good correlation exists between the Abbott 2 point-of-care analyzer results and reference laboratory tests for lipid levels and HbA1c (61, 62).

Cardiovascular risk factors and metabolic syndrome were defined according to the International Diabetes Federation (63). Markers and thresholds include abdominal obesity (waist circumference ≥ 80 cm for women or ≥ 94 cm for men), elevated fasting plasma triglycerides (>150 mg/dL), low HDL-C (<50 mg/dL for women or < 40 mg/dL for men), elevated fasting plasma glucose (>100 mg/dL) and hypertension (>130 mmHg systolic BP or > 85 mmHg diastolic BP). Elevated fasting plasma glucose was replaced with elevated HbA1c (>5.7%) (64). Metabolic syndrome is diagnosed if three or more criteria are fulfilled. The continuous metabolic syndrome score was calculated according to Eisenmann’s method (65). First, we standardized the individual cardiovascular risk markers for sex, age, and origin. Since HDL-C is associated with a reduction in metabolic risk, it was multiplied by−1. Finally, we calculated the sum of the standardized residuals to determine the metabolic syndrome score. A higher score indicates a worse metabolic syndrome profile.

Headache over the last week was measured with the Visual Analog Scale for Pain (VAS) (66). The VAS consists of a 100 mm horizontal line with two extremes 0 mm (no pain) and 100 mm (severe pain). Evidence of the validity of the VAS has been reported previously (67).

### Cardiorespiratory fitness

Cardiorespiratory fitness was measured with the (submaximal) Åstrand-Rhyming Indirect Test of Maximal Oxygen Uptake (68), performed on a bicycle ergometer. Maximal oxygen uptake (VO_2_max) was calculated based on sex, a correction factor for age, body weight, mean steady state, and power output (69). The validity of the Åstrand-Rhyming test for deriving VO_2_max has been documented previously (70). Sex and age-adjusted cut-offs distinguish between poor and fair or better cardiorespiratory fitness (71).

Perceived fitness was assessed with a 1-item fitness measure from 1 (poor fitness) to 10 (excellent fitness) (72). Previous studies showed that perceived fitness is moderately associated with objective fitness measures (73) and more closely with mental and physical health benefits (72).

### Statistical analyzes

Data was double-entered, checked, and merged into a single data file. Outliers were then detected using the Inter Quartile Range. After checking for outliers, one implausible value of 91.9 for cardiorespiratory fitness was removed. The individual mean score was used to impute missing values for calculating the total test score in the self-reported measures. Overall, few data were missing for mental (3%) and physical health (4%). Several missing values were detected for cardiorespiratory fitness (21%), mainly due to knee complaints or injuries. Frequencies (n, %) describe the study sample and prevalence, while mean score (M), standard deviation (SD) and confidence interval (95% CI) outline the severity of the outcome variables across predictors. We performed independent t-tests and chi-square tests to examine whether the final sample differs in the sociodemographic background from the broader screened eligible households. Additionally, chi-square tests were used to examine relationships between primary outcomes and predictors. To determine the association between the outcome and predictor variables, we conducted binary and multiple logistic regressions using the individual odds ratio (95% CI). The level of significance was set at *p* < 0.05 across all analyzes. Variables were included in multiple logistic regression analyzes when they were associated with the outcome variable at *p* < 0.10 in binary analysis. To reduce the number of variables in the regression models, we summarized the variables for severe depressive, severe anxiety and moderate insomnia symptoms in a factor termed “psychiatric symptoms.” Psychiatric symptoms were defined as scoring above the cut-off for a possible diagnosis of one or more mental disorders (depression, generalized anxiety disorder or insomnia). Sex, age, and origin were included in the multiple logistic regression models independently of their binary association with the outcome variable to control for sociodemographic background. Multicollinearity was checked after running the whole model. Statistical analyzes were performed with SPSS (Version 24, IBM, Armonk, United States) for both descriptive and inferential analysis.

## Results

### Sample

**Figure 1:**
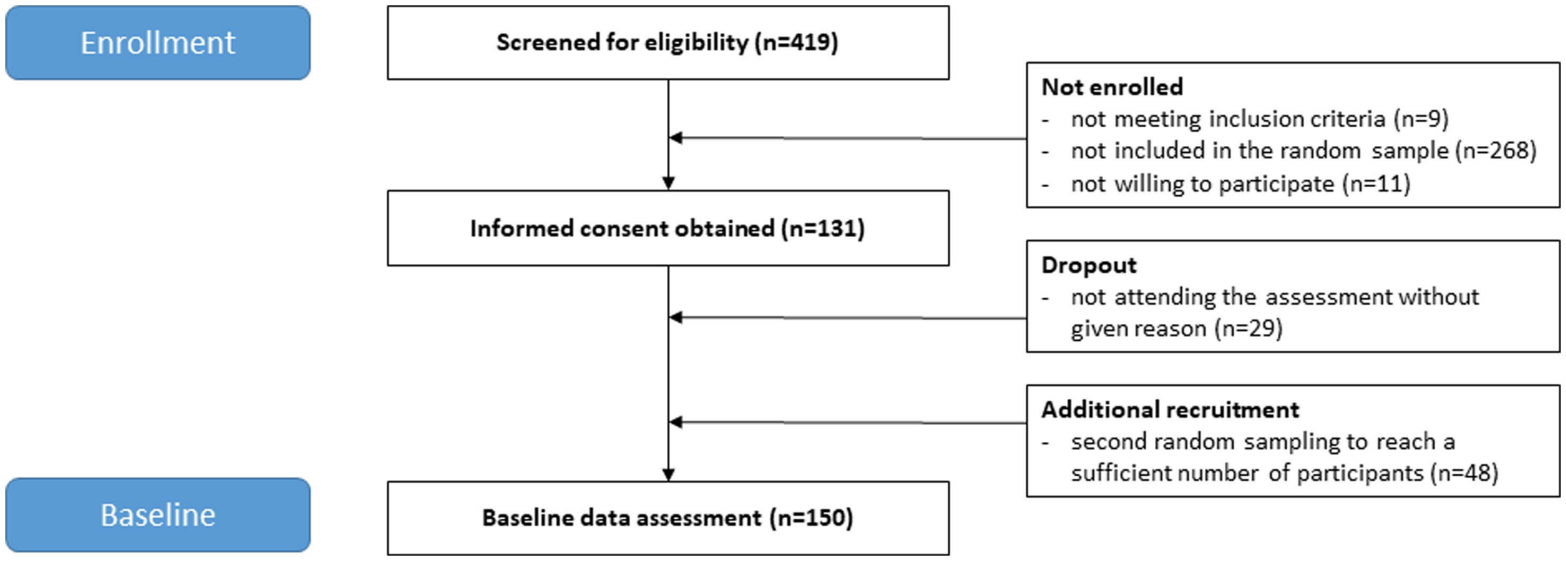
CONSORT flow diagram.

Information on participant flow is provided in Figure 1. The final sample consisted of 150 forcibly displaced individuals (75 female). The overall response rate was 79% (*n* = 190 invited individuals). Reasons for declining participation included illness, injury, refusal to take the legally required COVID-19 test, or reservations to leave the camp not to miss out on expected feedback from authorities. Characteristics of the study participants are presented in Table 1. The mean age of the total sample was 29.1 years (SD = 9.3). Most study participants were between 16 and 35 years old (*n* = 113, 79.0%), originated from Afghanistan (*n* = 79, 52.7%), and lived with one or more family members in the camp (*n* = 98, 66.2%). Overall, the mean stay in the camp was 14.8 months (SD = 10.2). Noticeable sex differences were the higher number of women without educational qualifications and the low number of university degrees (*n* = 2, 2.8%) compared to men (*n* = 18, 25.4%). Furthermore, no man in our sample had to care for a child alone, in contrast to the women (*n* = 11, 14.9%). After testing for group differences in sociodemographic background (age, origin, education, relatives in camp, time since flight and time in camp), the only difference found was that mean time since flight differed t(382.4) = 2.156, *p* = 0.032 between screened participants (*M* = 43.4, SD = 55.2) and the final sample (*M* = 33.4, SD = 36.5).

**Table 1:** Sociodemographic background of the study participants (*n* = 150).

Sociodemographics	Total *n* (%)	Female *n* (%)	Male *n* (%)
Age			
16–25	58 (40.6)	29 (39.7)	29 (41.4)
26–35	55 (38.5)	26 (35.6)	29 (41.4)
>35	30 (21.0)	18 (24.7)	12 (17.1)
Origin			
Sub-Sahara Africa	48 (32.0)	25 (33.3)	23 (30.7)
Southwest Asia	102 (68.0)	50 (66.7)	52 (69.3)
Education			
None	36 (25.5)	24 (33.8)	12 (17.1)
Primary	52 (36.9)	29 (40.8)	23 (32.9)
High school and above	53 (37.6)	18 (25.4)	35 (50.0)
Relatives in camp			
Alone	50 (33.8)	18 (24.3)	32 (43.2)
Relatives ≥1	98 (66.2)	56 (75.7)	42 (56.8)
Time since flight (months)			
0–24	62 (48.1)	32 (50.8)	30 (45.5)
>24	67 (51.9)	31 (49.2)	36 (54.5)
Time in camp (months)			
0–12	71 (51.4)	37 (53.6)	34 (49.3)
>12	67 (48.6)	32 (46.4)	35 (50.7)

### Psychological well-being

Summary statistics on psychological well-being across predictor variables are shown in Tables 2, 3. More than half of the participants scored above the cut-off for high psychological well-being. The mean score for psychological well-being was 53.9 (SD = 28.8) across the sample (*n* = 149, 1 missing). Descriptively, participants who were male, between 26 and 35 years old, or from Sub-Sahara Africa had higher mean scores in psychological well-being. However, differences were not statistically significant. In binary logistic regression analyzes, symptoms of depression, generalized anxiety disorder and insomnia, perceived stress, anemia, headache, and perceived fitness were associated with psychological well-being. There were no associations between psychological well-being, sociodemographic factors, and cardiorespiratory fitness. The results from the multiple logistic regression analysis of psychological well-being regressed on associated factors are shown in Table 4. Sex, age, origin, and all factors that were significantly associated with the outcome in the binary analyzes were included in the final model. In the final model, only psychiatric symptoms and perceived fitness remained significantly associated with psychological well-being. There is strong evidence that psychiatric symptoms were negatively (OR = 0.22, 95% CI 0.08–0.59, *p* = 0.003) and perceived fitness positively associated (OR = 1.35, 95% CI 1.11–1.63, *p* = 0.003) with high psychological well-being.

**Table 2:** Prevalence of mental distress and physical disorders in comparison with global mean.

	Total % (*n*)	Female % (*n*)	Male % (*n*)	Global mean %^b^
Mental health				
High psychological well-being (WHO-5)^a^	53.0 (79)	47.3 (35)	58.7 (44)	No reference
High PTSD symptoms (IES-R)^a^	35.3 (53)	44.0 (33)	26.7 (20)	3.9
Severe depressive symptoms (PHQ-9)^a^	33.3 (50)	41.3 (31)	25.3 (19)	12.0
Severe anxiety symptoms (GAD-7)^a^	27.9 (39)	38.5 (25)	18.7 (14)	16.0
Moderate insomnia symptoms (ISI)^a^	33.8 (47)	37.5 (24)	30.7 (23)	33.3
High perceived stress (PSS-10)^a^	52.2 (72)	65.6 (42)	40.5 (30)	No reference
Physical health				
Metabolic syndrome^a^	28.8 (40)	30.0 (21)	27.5 (19)	25.0
Abdominal obesity^a^	48.0 (71)	71.2 (52)	25.3 (19)	41.5
Dyslipidemia^a^	22.7 (30)	19.1 (13)	26.6 (17)	No reference
Hypertension^a^	38.9 (58)	21.6 (16)	56.0 (42)	31.1
Prediabetes^a^	13.1 (19)	13.7 (10)	12.5 (9)	15.3
Anemia^a^	27.3 (39)	31.9 (23)	22.5 (16)	23.7
High headache (VAS)^a^	47.8 (66)	56.3 (36)	40.5 (30)	No reference
Fitness				
Fair and higher VO_2_max^a^	24.6 (29)	18.9 (10)	29.2 (19)	No reference
High perceived fitness^a^	55.9 (76)	42.2 (27)	68.1 (49)	No reference

^a^Cut-off: WHO-5 (≥50), IES-R (≥46), PHQ-9 (≥15), GAD-7 (≥15), ISI (≥15), PSS-15 (≥median), metabolic syndrome (≥3 out of 5), abdominal obesity (waist circumference ≥ 80 cm for women or ≥ 94 cm for men), dyslipidemia (fasting plasma triglycerides > 150 mg/d and HDL-C < 50 mg/dL for women or < 40 mg/dL for men), hypertension (>130 mmHg systolic BP or > 85 mmHg diastolic BP), prediabetes (HbA1c = 5.7–6.4%), anemia (women < 120 g/L or men < 130 g/L), VAS (≥median), VO_2_max [based on ACSM guidelines (71)], perceived fitness (≥median).

^b^According to the literature (74–81).

**Table 3:** Descriptive statistics of psychological well-being (WHO-5) dichotomized into high vs. low well-being.

	Index Score	High (≥50)	Low (<50)	*χ* ^2^
	*M* (95% CI)	*n* (%)	*n* (%)	
Sociodemographics				
Sex				0.164
Female	50.7 (43.9–57.5)	35 (47.3)	39 (52.7)	
Male	57.0 (50.5–63.5)	44 (58.7)	31 (41.3)	
Age				0.174
16–25	51.3 (43.9–58.7)	30 (51.7)	28 (48.3)	
26–35	59.2 (51.3–67.1)	33 (61.1)	21 (38.9)	
>35	47.2 (36.6–57.7)	12 (40.0)	18 (60.0)	
Origin				0.073
Sub-Sahara Africa	57.6 (48.8–66.5)	30 (63.8)	17 (36.2)	
Southwest Asia	52.1 (46.6–57.7)	49 (48.0)	53 (52.0)	
Education				0.828
None	54.6 (44.4–64.7)	18 (50.0)	18 (50.0)	
Primary	51.0 (43.0–58.9)	25 (49.0)	26 (51.0)	
High school and above	53.3 (45.7–60.8)	29 (54.7)	24 (45.3)	
Relatives in camp				0.149
Alone	55.7 (46.9–64.5)	31 (62.0)	19 (38.0)	
Relatives ≥1	53.7 (48.1–59.2)	48 (49.5)	49 (50.5)	
Time since flight (months)				0.358
0–24	55.1 (47.7–62.5)	35 (57.4)	26 (42.6)	
>24	51.9 (45.1–58.8)	33 (49.3)	34 (50.7)	
Time in camp (months)				0.673
0–12	52.2 (45.8–58.7)	37 (52.9)	33 (47.1)	
>12	52.6 (45.2–59.9)	33 (49.3)	34 (50.7)	
Mental health				
Severe depressive symptoms (PHQ-9)^a^				<0.001
Yes	35.7 (29.5–41.9)	11 (22.4)	38 (77.6)	
No	62.8 (57.2–68.3)	68 (68.0)	32 (32.0)	
Severe anxiety symptoms (GAD-7)^a^				<0.001
Yes	33.9 (27.3–40.5)	8 (20.5)	31 (79.5)	
No	58.5 (52.9–64.1)	61 (61.0)	39 (39.0)	
Moderate insomnia symptoms (ISI)^a^				<0.001
Yes	38.9 (31.8–46.1)	12 (26.1)	34 (73.9)	
No	57.4 (51.6–63.3)	56 (60.9)	36 (39.1)	
High perceived stress (PSS-10)^a^				<0.001
Yes	43.4 (37.4–49.4)	26 (36.6)	45 (63.4)	
No	59.5 (52.3–66.6)	41 (62.1)	25 (37.9)	
Physical health				
Anemia^a^				0.047
Yes	48.9 (39.0–58.8)	16 (41.0)	23 (59.0)	
No	57.0 (51.6–62.5)	62 (59.6)	42 (40.4)	
High headache (VAS)^a^				0.072
Yes	45.7 (38.9–52.5)	27 (41.5)	38 (58.5)	
No	56.4 (49.8–63.1)	41 (56.9)	31 (43.1)	
Fitness				
VO_2_max^a^				0.991
Fair and above	57.8 (46.2–69.4)	16 (55.2)	13 (44.8)	
Poor and below	52.0 (46.2–57.8)	49 (55.1)	40 (44.9)	
High perceived fitness^a^				<0.001
Yes	60.0 (53.6–66.4)	48 (63.2)	28 (36.8)	
No	39.1 (33.0–45.1)	18 (30.5)	41 (69.5)	

^a^Cut-off: PHQ-9 (≥15), GAD-7 (≥15), ISI (≥15), PSS-10 (≥median), VAS (≥median), anemia (women ≤ 120 g/L or men ≤ 130 g/L), VO_2_max [based on ACSM guidelines (71)], perceived fitness (≥median).

**Table 4:** Logistic models of high psychological well-being (WHO-5), high PTSD symptoms (IES-R), and metabolic syndrome regressed on sex, age, origin, and associated factors.

	Model high psychological well-being (≥50)^a^			Model high PTSD symptoms (≥46)^b^			Model metabolic syndrome (≥3 out of 5)^c^		
	OR	95% CI	*p*-value	OR	95% CI	*P*-value	OR	95% CI	*P*-value
Sociodemographics									
Sex									
Female	Reference			Reference			Reference		
Male	0.88	(0.32–2.45)	0.802	0.77	(0.29–2.07)	0.608	3.27	(0.90–11.97)	0.073
Age	1.00	(0.95–1.05)	0.916	1.00	(0.95–1.05)	0.951	1.02	(0.96–1.09)	0.496
Origin									
Sub-Sahara Africa	Reference			Reference			Reference		
Southwest Asia	0.76	(0.28–2.12)	0.604	1.99	(0.48–8.29)	0.345	4.02	(1.02–15.77)	0.046
Relatives in camp									
Alone				Reference					
Relatives 1 ≤				0.70	(0.17–2.91)	0.623			
Mental health									
Psychiatric symptoms^d^									
No	Reference			Reference					
Yes	0.22	(0.08–0.59)	0.003	3.27	(1.10–9.74)	0.034			
Perceived stress (PSS-10)	1.00	(0.93–1.06)	0.923	1.13	(1.05–1.23)	0.002			
Physical health									
Hemoglobin	1.02	(0.99–1.06)	0.184						
Headache	1.00	(0.98–1.02)	0.977	1.01	(1.00–1.03)	0.136			
Fitness									
VO_2_max							0.92	(0.86–0.99)	0.021
Perceived fitness	1.35	(1.11–1.63)	0.003	0.93	(0.76–1.13)	0.453	0.80	(0.65–0.98)	0.031
Nagelkerkes *R*^2^	0.36			0.47			0.20		

^a^Includes the variables sex, age, origin, psychiatric symptoms, perceived stress, hemoglobin, headache and perceived fitness.

^b^Includes the variables sex, age, origin, relatives in camp, psychiatric symptoms, perceived stress, headache and perceived fitness.

^c^Includes the variables sex, age, origin, VO2max and perceived fitness.

^d^Psychiatric symptoms is defined as having elevated symptoms in one or more mental disorder: PHQ-9 (≥15), GAD-7 (≥15), or ISI (≥15).

### Mental distress

The prevalence of mental distress and high PTSD symptoms across sociodemographic and clinical variables are shown in Tables 2, 5. One out of three participants scored above the clinical cut-off for PTSD, depression, generalized anxiety disorder, and insomnia. Fifty-five (39.8%) participants had considerably elevated symptoms in at least one or more psychiatric disorders. The mean severity score for PTSD was 34.6 (SD = 22.4) in the whole sample (*n* = 150). Higher PTSD scores were found in female participants aged older than 35 years, originating from Southwest Asia, or living with one or more relatives in the camp. Notably, sex, origin, and living with one or more relatives in the camp were significantly associated with PTSD symptoms in binary logistic regression analyzes besides symptoms of depression, generalized anxiety disorder and insomnia, perceived stress, headache, and perceived fitness. On the contrary, education, time spent in the camp, anemia, and cardiorespiratory fitness were not associated with PTSD symptom severity. The final model of the multiple logistic regression analysis, including age and all associated factors from binary analyzes, is presented in Table 4. In the final model, psychiatric symptoms (OR = 3.27, 95% CI 1.10–9.74, *p* = 0.034) and perceived stress (OR = 1.13, 95% CI 1.05–1.23, *p* = 0.002) were associated with the occurrence of high PTSD severity.

**Table 5:** Descriptive statistics of PTSD (IES-R) dichotomized into high vs. low PTSD symptom severity.

	Index Score	High (≥46)	Low (<46)	*χ* ^2^
	*M* (95% CI)	*n* (%)	*n* (%)	
Sociodemographics				
Sex				0.026
Female	39.0 (33.8–44.1)	33 (44.0)	42 (56.0)	
Male	30.3 (25.3–35.4)	20 (26.7)	55 (73.3)	
Age				0.379
16–25	31.6 (25.6–37.6)	17 (29.3)	41 (70.7)	
26–35	36.2 (30.8–41.6)	23 (41.8)	32 (58.2)	
>35	39.1 (29.8–48.3)	11 (36.7)	19 (63.3)	
Origin				0.011
Sub-Sahara Africa	29.3 (23.0–35.4)	10 (20.8)	38 (79.2)	
Southwest Asia	37.2 (32.8–41.6)	43 (42.2)	59 (57.8)	
Education				0.107
None	36.8 (29.6–43.9)	14 (38.9)	22 (61.1)	
Primary	39.1 (32.5–45.6)	24 (46.2)	28 (53.8)	
High school and above	31.6 (25.7–37.5)	14 (26.4)	39 (73.6)	
Relatives in camp				0.012
Alone	28.7 (22.9–34.4)	11 (22.0)	39 (78.0)	
Relatives ≥1	37.9 (33.3–42.5)	42 (42.9)	56 (57.1)	
Time since flight (months)				0.743
0–24	34.8 (29.3–40.4)	23 (37.1)	39 (62.9)	
>24	34.7 (29.3–40.2)	23 (34.3)	44 (65.7)	
Time in camp (months)				0.933
0–12	36.0 (30.9–41.2)	26 (36.6)	45 (63.4)	
>12	35.7 (30.1–41.2)	25 (37.3)	42 (62.7)	
Mental health				
Severe depressive symptoms (PHQ-9)^a^				<0.001
Yes	49.8 (44.2–55.5)	33 (66.0)	17 (34.0)	
No	27.1 (23.2–31.0)	20 (20.0)	80 (80.0)	
Severe anxiety symptoms (GAD-7)^a^				<0.001
Yes	56.1 (50.9–61.4)	29 (74.4)	10 (25.6)	
No	28.0 (24.1–31.9)	24 (23.8)	77 (76.2)	
Moderate insomnia symptoms (ISI)^a^				<0.001
Yes	50.4 (44.9–55.9)	29 (61.7)	18 (38.3)	
No	28.6 (24.3–32.9)	24 (26.1)	68 (73.9)	
High perceived stress (PSS-10)^a^				<0.001
Yes	46.5 (41.7–51.2)	43 (59.7)	29 (40.3)	
No	25.0 (20.1–30.0)	10 (15.2)	56 (84.8)	
Physical health				
Anemia^a^				0.226
Yes	39.1 (31.5–46.7)	17 (43.6)	22 (56.4)	
No	33.7 (29.5–37.9)	34 (32.7)	70 (67.3)	
High headache (VAS)^a^				<0.001
Yes	45.9 (40.3–51.4)	37 (56.1)	29 (43.9)	
No	27.0 (22.5–31.4)	16 (22.2)	56 (77.8)	
Fitness				
VO_2_max ^a^				0.138
Fair and above	31.9 (24.2–39.6)	7 (24.1)	22 (75.9)	
Poor and below	35.4 (30.5–40.3)	35 (39.3)	54 (60.7)	
High perceived fitness^a^				0.019
Yes	31.1 (26.2–36.1)	23 (30.3)	53 (69.7)	
No	42.8 (37.1–48.6)	30 (50.0)	30 (50.0)	

^a^Cut-off: PHQ-9 (≥15), GAD-7 (≥15), ISI (≥15), PSS-10 (≥median), VAS (≥median), anemia (women ≤ 120 g/L or men ≤ 130 g/L), VO2 max [based on ACSM guidelines (71)], perceived fitness (≥median).

### Physical health

The summary statistics on the prevalence of cardiovascular risk factors, anemia, high level of headache, and metabolic syndrome across predictors are shown in Tables 2, 6. Every fourth participant (*n* = 139, 11 missing) met the inclusion criteria for metabolic syndrome, and three out of four women scored above the cut-off for abdominal obesity. Apart from age, cardiorespiratory and perceived fitness, no other factor was associated with metabolic syndrome in the binary analyzes. The results of the final multiple logistic regression model are presented in Table 4. After including sex, origin, and all associated factors in the regression, only origin, cardiorespiratory and perceived fitness remained significantly associated with metabolic syndrome. Individuals from Southwest Asia (OR = 4.02, 95% CI 1.02–15.77, *p* = 0.046) were more likely to be affected with metabolic syndrome, whereas participants with higher levels of objectively measured (OR = 0.92, 95% CI 0.86–0.99, *p* = 0.021) and subjectively perceived fitness (OR = 0.80, 95% CI 0.65–0.98, *p* = 0.031) were less likely to be affected with metabolic syndrome.

**Table 6:** Descriptive statistics of metabolic syndrome dichotomized into present vs. absent metabolic syndrome.

	Index score^b^	Yes (≥3 out of 5)	No (<3 out of 5)	*χ* ^2^
	*M* (95% CI)	*n* (%)	*n* (%)	
Sociodemographics				
Sex				0.748
Female	0.05 (−0.66–0.76)	21 (30.0)	49 (70.0)	
Male	0.16 (−0.47–0.78)	19 (27.5)	50 (72.5)	
Age				0.001
16–25	0.50 (−0.16–1.16)	9 (17.0)	44 (83.0)	
26–35	−0.33 (−0.99–0.32)	14 (26.9)	38 (73.1)	
>35	0.20 (−1.27–1.66)	15 (55.6)	12 (44.4)	
Origin				0.171
Sub-Sahara Africa	−0.06 (−0.89–0.77)	9 (20.9)	34 (79.1)	
Southwest Asia	0.16 (−0.41–0.73)	31 (32.9)	65 (67.7)	
Education				0.185
None	−0.12 (−0.95–0.72)	6 (17.1)	29 (82.9)	
Primary	0.00 (−0.90–0.89)	13 (27.7)	34 (72.3)	
High school and above	0.32 (−0.44–1.08)	17 (35.4)	31 (64.6)	
Relatives in camp				0.582
Alone	0.37 (−0.41–1.16)	12 (25.5)	35 (74.5)	
Relatives ≥1	0.02 (−0.58–0.61)	27 (30.0)	63 (70.0)	
Time since flight (months)				0.994
0–24	0.10 (−0.63–0.83)	14 (25.5)	41 (74.5)	
>24	0.09 (−0.59–0.76)	16 (25.4)	47 (74.6)	
Time in camp (months)				0.209
0–12	−0.19 (−0.84–0.47)	20 (31.7)	43 (68.3)	
>12	0.30 (−0.43–1.02)	14 (21.9)	50 (78.1)	
Mental health				
Severe depressive symptoms (PHQ-9)^a^				0.271
Yes	−0.03 (−0.89–0.83)	16 (34.8)	30 (65.2)	
No	0.17 (−0.40–0.74)	24 (25.8)	69 (74.2)	
Severe anxiety symptoms (GAD-7)^a^				0.074
Yes	0.18 (−0.82–1.18)	15 (40.5)	22 (59.5)	
No	0.05 (−0.53–0.62)	23 (24.7)	70 (75.3)	
Moderate insomnia symptoms (ISI)^a^				0.165
Yes	0.34 (−0.41–1.10)	16 (36.4)	28 (63.6)	
No	−0.12 (−0.77–0.52)	21 (24.7)	64 (75.3)	
High perceived stress (PSS-10)^a^				0.961
Yes	−0.22 (−0.94–0.49)	19 (27.9)	49 (72.9)	
No	0.32 (−0.36–1.01)	17 (28.3)	43 (71.7)	
Physical health				
Anemia^a^				0.485
Yes	0.04 (−1.01–1.10)	9 (24.3)	28 (75.7)	
No	0.12 (−0.41–0.65)	31 (30.4)	71 (69.6)	
High headache (VAS)^a^				0.674
Yes	−0.13 (−0.87–0.62)	19 (30.6)	43 (69.4)	
No	0.25 (−0.41–0.91)	18 (27.3)	48 (72.7)	
Fitness				
VO_2_max^a^				0.005
Fair and above	−0.61 (−1.52–0.30)	1 (3.7)	26 (96.3)	
Poor and below	0.13 (−0.41–0.68)	25 (30.1)	58 (69.9)	
High perceived fitness^a^				0.044
Yes	−0.35 (−0.92–0.23)	15 (20.8)	57 (79.2)	
No	0.48 (−0.37–1.33)	20 (37.0)	34 (63.0)	

^a^Cut-off: PHQ-9 (≥15), GAD-7 (≥15), ISI (≥15), PSS-10 (≥median), VAS (≥median), anemia (women ≤ 120 g/L or men ≤ 130 g/L), VO2 max [based on ACSM guidelines (71)], perceived fitness (≥median).

^b^Index score is composed of continuous metabolic syndrome score.

## Discussion

This study extends existing findings on health challenges of forcibly displaced people by assessing the psychological well-being, PTSD symptom severity, metabolic syndrome, and associated factors among people living in a refugee camp in Greece.

Only half of the study participants rated their psychological well-being as high. In comparison, forcibly displaced adults resettled in Sweden scored 7.1% (*M* = 57.7, SD = 27.1), and the broader population in Greece during the first year of the COVID-19 pandemic 23.4% (*M* = 66.5, SD = 22.7) higher (82, 83). Moreover, consistent with previous findings (7, 9, 10, 14, 53, 84, 85), there is a considerably elevated prevalence of mental and physical distress among forcibly displaced people in Greece. Compared to the global mean (74–81), the risk of being affected by PTSD is nine, depression three, and generalized anxiety disorder one and a half times higher among forcibly displaced people. No difference in prevalence was found for moderate or severe insomnia symptoms, metabolic syndrome, abdominal obesity, hypertension, prediabetes, and anemia. Our findings stand in contradiction to a recent large-scale study (86), which collected data at health clinics in Southern European reception centers and reported a prevalence of 0.7% for PTSD and 5.7% for cardiovascular disease. As highlighted by the authors, the discrepant results may be explained by an inadequate recording of health conditions by health professionals. In addition, low mental health help-seeking behavior has been found in this population. Constrained health services, limited health literacy, lack of trust in authorities or cultural barriers such as mental health stigmas could have discouraged individuals from voluntarily seeking a health check and lead to a higher number of unreported cases (87). Another reason for the conflicting results might be the different sociodemographic background of the participants, who, in contrast to our study, were predominantly male (77.7%) and without relatives in the camp (80.3%).

Forcibly displaced people represent a heterogeneous group with different personal traits, sociodemographic backgrounds, and lived experiences. Therefore, the question arises if individuals with specific characteristics are more strongly affected by certain conditions than others. Subgroup analyzes did not identify a specific target group with high mental well-being. However, female Southwest Asian people living with family members in the camp had higher PTSD symptom scores. The differences between sexes have been explained in previous studies (9, 84, 88), with women being at higher risk of sexual violence, childcare pressures, and exploitation. Unlike other results (89), being together with a family member was associated with higher PTSD symptom scores in our sample. This finding may be surprising, as social support has been shown to have a buffering effect against PTSD (90). Possible explanations for this could be that the family does not necessarily provide social support in the face of family conflict and violence (5). Moreover, caring for the whole family can pose an additional challenge and may trigger negative mental states. The life experience that caused the entire family to flee their country rather than as individuals may also explain the difference. However, these remain assumptions and should be further investigated in larger studies with increased statistical power and additional qualitative methods to identify subgroup differences and possible rationales.

Regarding cardiovascular risk factors, older age and origin from Southwest Asia were associated with metabolic syndrome. Similarly, a recent meta-analysis (10) reported a higher risk of cardiovascular disease among older forcibly displaced adults from the Middle East. The elevated risk has been attributed to psychological stress and acculturation processes such as changes in lifestyle and diet (7, 8). In our study, neither psychiatric symptoms nor time spent in the camp were associated with the occurrence of metabolic syndrome. The difference may be due to the fact that the two studies examined migrating populations in general and not exclusively forcibly displaced people who spent a shorter time in the host country. Living in the camp instead of within society may also have led to no or limited acculturation processes. Furthermore, the prevalence of the metabolic syndrome is generally significantly higher in the Eastern Mediterranean Region (91), suggesting that the increased risk is more related to the risk profile of the region of origin than to the specific experience of forced displacement. Overall, the associations between sociodemographic background and mental and physiological outcomes, except for Asian origin, lose statistical significance in multivariable analyzes when the clinical factors are considered.

When living in a precarious situation, time itself does not seem to support recovery from PTSD symptom severity. To date, the mitigating effect of time on PTSD is controversially discussed and ranges from improvement to no effect till exacerbation of PTSD symptomatology (85, 86, 90). Different exposure to post-migration living difficulties could explain the contrasting results. In our study, perceived stress was associated with the occurrence of higher PTSD symptoms, while no relationship between the length of stay and PTSD symptoms was detected. These observations support recent findings (92) that post-migration stressors maintain the severity of PTSD symptoms. The decisive factors are ongoing daily stressors, especially lengthy asylum procedures, uncertain visa status, detention in refugee camps, or legal barriers to work (93).

Particularly noteworthy is that people who perceived their fitness level as higher had better overall health scores. Surprisingly, this did not apply to objectively measured cardiorespiratory fitness, which was associated only with metabolic syndrome. The association between perceived fitness and psychological well-being and metabolic syndrome remained statistically significant after controlling for confounding factors. This suggests that mental health is more closely associated with an individual’s perceived subjective fitness than objective fitness measurement. As such, the process of meaning-making in relation to personal fitness appears to be of particular importance. Our findings add to the small body of knowledge, suggesting that perceived fitness is associated with both mental and physical health benefits (72, 94), pointing out that cognitive factors should be taken into account when studying the effect of sport and exercise activities implemented in a refugee camp. Importantly in the context of a refugee camp, perceived fitness was more strongly associated with psychological functioning and daily coping than cardiorespiratory fitness in a sample of psychology students (73). Furthermore, participants with high perceived fitness scored lower on insomnia, perceived higher sleep quality, and ruminated less about unresolved problems than participants who rated their fitness low (95). This last point requires particular attention because “thinking too much” expresses emotional and cognitive distress in certain Sub-Saharan and Southwest Asian cultures. For forcibly displaced people in a protracted situation, repetitive negative thinking can exacerbate psychological, physical, and social symptoms (96). People with high perceived fitness may be less affected by the cognitive loop about current life situations and past events, which may lead to higher overall well-being.

In line with the current findings of an increased risk for mental distress compared to the global population and the overall high prevalence of mental and physiological distress among people living in a refugee camp in Greece, organizations on-site criticize the lack of early measures (97). The lack of prevention or early treatment favors the development or consolidation of mental disorders and undermines the achievement of the health-related United Nations Sustainable Development Goals by 2030. Based on the results of this and previous studies (4, 5, 15, 92, 93), policies are urgently needed to reduce post-migration stressors and prevent mental health deterioration in Greek refugee camps. Prevention should be of priority, complemented by targeted programs to address population-based health needs and psychological well-being. The extent of diverse health needs and the heterogeneous composition of the population suggests that general health support should go beyond medical treatment and include a set of complementary measures to balance health inequities. These include access to education, a perspective on economic independence, social services, community capacity strengthening, health literacy promotion, and psychosocial activities. In light of the present findings, organized sport and exercise activities might be a favorable add-on intervention to address mental and physiological health.

While sport and exercise activities have successfully been implemented to address mental and physical health (12, 98–100), evidence for similar effects among forcibly displaced people is scarce (101). As these are substantially different contexts, future studies need to investigate the effect and feasibility of regular sport and exercise activities on mental and physiological health among people living in refugee camps. In addition, this study’s results raise the question of whether improvements in perceived fitness are associated with improvements in mental and physical health and, which and how activities should be implemented to address perceived fitness.

### Strengths and limitations

This study has certain strengths, such as providing a comprehensive perspective of health and its associated factors among forcibly displaced people living in a refugee camp in Greece. Given the recent developments in forcible displacement and since Greece is one of the main entry points in Europe, an essential contemporary context was studied. Furthermore, we addressed a population that is difficult to reach due to challenging living situations and restricted camp access. Our study is, to our knowledge, one of the first which examines the association of perceived fitness and mental and physiological health among forcibly displaced people within a refugee camp context. Lastly, the study applied methodological rigorousness by following a pre-registered study protocol (20). The shortage of sex-specific results (88) and the barriers to accessing health services, in particular mental health (87), was addressed by a random and stratified sample by sex, including the most represented ethnic groups in the camp.

Limitations include the cross-sectional nature of the present data and the small-to-moderate sample size, increasing the risk that minor associations were overseen. On the other hand, multiple testing might have increased the occurrence of Type I errors. Despite an acceptable response rate, one might assume that selection bias influenced the results. However, when analyzing group differences, only time since flight differed statistically significantly from the broader screened camp population. Generalizability to other settings might be limited, as post-migration life circumstances differ from context to context and significantly impact overall health (4). Expanding the study to other camps, particularly those on the Greek islands, which have been described as more restrictive (97), could therefore provide valuable insights to complement our findings. A key finding of the study was that cardiorespiratory fitness, unlike perceived fitness, was not associated with mental health outcomes. The considerable amount of missing values for cardiorespiratory fitness may have affected this finding. Moreover, recent literature suggests that self-report symptom-based measures tend to overestimate the prevalence of mental distress (52–54). This may also be caused by participants indicating higher initial values in the hope that this will positively influence their asylum procedure (89). These circumstances were addressed by information about study intentions and the use of more conservative, clinically relevant cut-offs. Validated instruments widely used in different cultural contexts have been implemented, though none have been developed explicitly for cross-cultural use (84). The universality of Western-based classification systems to describe mental distress in other contexts is accordingly questioned by some authors (52, 53, 84). Similarly, population-specific cut-offs for cardiovascular risk factors have been recommended (102). The International Diabetes Federation advocates ethnicity-specific cut-offs for central obesity (63). To date, however, these are not available for Sub-Saharan and Eastern Mediterranean populations. Instead, European reference values had to be used, which might lead to inaccuracies in our results.

## Conclusion

The findings of this study underpin the call for urgent action to address the health needs of people living in refugee camps in Greece. Compared to the global population, forcibly displaced people in Greece have a considerably higher risk of being affected by mental distress. The prevalence of moderate or severe insomnia symptoms and physiological disorders is likewise elevated. However, they do not differ from the global mean. Therefore, the first priority should be to prevent the deterioration of health conditions by reducing post-migration stress. Second, population-centered programs must be developed to address mental health and non-communicable diseases. Sport and exercise programs could be a favorable adjunct, as perceived fitness, as a potentially protective factor, is associated with mental and physiological health benefits.

## Data availability statement

The raw data supporting the conclusions of this article will be made available by the authors, without undue reservation.

## Ethics statement

The studies involving human participants were reviewed and approved by Research Ethics Committee of the University of Thessaly. The patients/participants provided their written informed consent to participate in this study.

## Author contributions

AH, IM, and MG supervised the study. FK and KF coordinated the fieldwork. FK analyzed the data and drafted the initial manuscript. KF, AH, IM, ET, EH, HS, FC, SL, MM, DQ, YT, RK, UP, and MG contributed to revising and editing the final manuscript. All authors were involved in the design of the study or contributed to the refinement of methods and read and approved the final manuscript.

## Funding

This study was partly funded by the Swiss Network for International Studies (SNIS). The funding source had no influence on the design of the study, the collection, management, analysis, and interpretation of the data, the writing of the manuscript, or on the selection of the journal.

## Conflict of interest

The authors declare that the research was conducted in the absence of any commercial or financial relationships that could be construed as a potential conflict of interest.

## Publisher’s note

All claims expressed in this article are solely those of the authors and do not necessarily represent those of their affiliated organizations, or those of the publisher, the editors and the reviewers. Any product that may be evaluated in this article, or claim that may be made by its manufacturer, is not guaranteed or endorsed by the publisher.

## Abbreviations

95% CI: 95% Confidence interval
BMI: Body mass index; BP: Blood pressure
DSM-5: Diagnostic and Statistical Manual of Mental Disorders (5th edition)
GAD-7: Generalized Anxiety Disorder scale (7-item version)
Hb: Hemoglobin
HbA1c: Glycated hemoglobin
HDL-C: High-density lipoprotein cholesterol
ICD-10: International Classification of Diseases (10th version)
IES-R: Impact of Event Scale-Revised; ISI: Insomnia Severity Index
ISRCTN: trial registry Primary clinical trial registry recognized by the WHO and ICMJE
M: Mean: OR: Odds ratio
PHQ-9: Patient Health Questionnaire (9-item version)
PSS-10: Perceived Stress Scale (10-item version)
PTSD: Post-traumatic stress disorder
SD: Standard deviation
SPSS: Statistical Package for the Social Sciences
VAS: Visual analog scale
VO2max (ml/kg/min): Maximal oxygen uptake
WHO: World Health Organization
WHO-5: 5-item World Health Organization Well-Being Index
